# Influence of the Culture Medium in Dose-Response Effect of the Chlorhexidine on* Streptococcus mutans* Biofilms

**DOI:** 10.1155/2016/2816812

**Published:** 2016-05-11

**Authors:** Vanessa Salvadego de Queiroz, Renzo Alberto Ccahuana-Vásquez, Alcides Fabiano Tedesco, Luzia Lyra, Jaime Aparecido Cury, Angélica Zaninelli Schreiber

**Affiliations:** ^1^Department of Clinical Pathology/School of Medical Sciences, FCM, State University of Campinas (UNICAMP), P.O. Box 6111, 13083-970 Campinas, SP, Brazil; ^2^Procter and Gamble Co., Frankfurter Straße 145, 61476 Kronberg im Taunus, Germany; ^3^São Leopoldo Mandic, School of Dentistry and Research Center, Rua José Rocha Junqueira, 13 Ponte Preta, 13045-755 Campinas, SP, Brazil; ^4^Department of Physiological Sciences, Piracicaba Dental School, FOP, State University of Campinas (UNICAMP), P.O. Box 52, 13414-903 Piracicaba, SP, Brazil

## Abstract

The aim of this study was to evaluate the influence of culture medium on dose-response effect of chlorhexidine (CHX) on* Streptococcus mutans* UA159 biofilm and validate the use of the cation-adjusted-Müller-Hinton broth (MH) for the evaluation of antibacterial activity. Ultrafiltered Tryptone-Yeast Extract Broth (UTYEB) was compared against MH and MH with blood supplementation (MHS). For each medium, six groups (*n* = 4) were assessed: two negative control groups (baseline 48 and 120 h) and four experimental groups (0.0001, 0.001, 0.012, and 0.12% CHX).* S. mutans* biofilm grew on glass slides of each media containing 1% sucrose. After 48 h of growth, biofilms of baseline 48 h were collected and the other groups were treated for 1 min, twice a day, for 3 days, with their respective treatments. The media were changed daily and pH was measured. After 120 h, biofilms were collected and dry weight and viable microorganisms were determined. Results showed CHX dose-response effect being observed in all media for all the variables. However, MH and MHS showed higher sensitivity than UTYEB (*p* < 0.05). We can conclude that the culture medium does influence dose-response effect of CHX on* Streptococcus mutans* biofilm and that MH can be used for antibacterial activity.

## 1. Introduction

*Streptococcus mutans,* primary etiological agent of dental caries in animals and humans, is also involved in biofilm formation and accumulation [1]. It is considered the most implicated microorganism in dental caries [2, 3] as it presents acidogenic and aciduric properties as well as having the ability to survive, grow, and maintain its metabolism under acidic conditions [4]. Therefore,* S. mutans* biofilms have been used in* in vitro* tests to evaluate cariogenic properties due to difficulties of developing* in vivo* studies for controlled cariogenic situations [5]. This microorganism is able to produce extracellular polysaccharide (EPS) from dietary carbohydrates, especially sucrose, that has been considered the most cariogenic carbohydrate [6], once it is the main substrate of cariogenic bacteria to synthesize EPS [7]. Extracellular polysaccharides improve bacterial adherence to tooth surfaces and modifies the biofilm matrix [8], increasing the porosity of dental biofilm matrix by the presence of these insoluble glucans [9, 10], facilitating the installation of caries disease [11, 12] and the shift in biofilm microbiota induced by pH fall [13], resulting in equilibrium disturbance of biofilm and tooth.

Chlorhexidine (CHX) is the most studied and effective antimicrobial agent in the chemical control of dental plaque, being considered the positive control (gold standard), to which all other antiplaque agents should be compared to [14]. It is a cationic bis-biguanide, with a wide antibacterial activity, low mammalian cells toxicity, and a high affinity to attach to skin and mucous membranes. Its mechanism of action includes direct damage to the internal cytoplasmatic membrane, being bacteriostatic at low doses and bactericidal at high concentrations. Its advantages are not only based on its antimicrobial properties but also on its affinity to attach to a wide variety of substrates. This property, known as substantivity, allows this compound to attain effective antibacterial levels, using a reasonable dosage (twice a day), thus allowing patients to comply with its use [15].

The potential of oral antimicrobials was usually evaluated in classical Minimum Inhibitory Concentration (MIC) and Minimum Bactericidal Concentration (MBC) tests, using planktonic monocultures and prolonged exposure to mouthrinses. In comparison with clinical tests the resulting inhibitory concentrations were 100–1000 times lower [16]. However, bacteria growing as a biofilm on a surface show reduced sensitivity to killing by antimicrobials, especially in older (more mature) biofilms. The reasons for this vary among inhibitors but include (a) reduced penetration of the agent, for example, due to binding to the biofilm matrix or quenching of the agent at biofilm surface, (b) the novel phenotype expressed by bacteria when growing on a surface, and (c) the slow growth rates of attached bacteria within biofilms [17].

Thus, they allowed only relative comparisons and were poorly predictive of the clinical efficacy of antiseptics.* In vitro* studies of dental biofilms models have been developed to mimic what occurs in the oral environment. However, there is not, in literature, a standardization regarding the used culture medium, which can be relevant to determine the relation dose-effect antimicrobial activity. Conversely, the nutrient medium content was found to regulate the development of biofilms in several organisms [18–20]. Therefore, the aim of this study was to evaluate the influence of culture medium on dose-response effect of the chlorhexidine, gold standard, on* S. mutans* biofilm model using cation-adjusted-Müller-Hinton broth (MH) medium, as indicated by CLSI M7-A6 [21] for planktonic cells, with or without lysed horse blood [21], to validate the use of the MH culture media.

## 2. Material and Methods

### 2.1. Experimental Design

This* S. mutans* biofilm model was a modified version detailed by Koo et al. [22] and Ccahuana-Vásquez and Cury [23], using culture medium and inoculum prepared as indicated by CLSI M7-A6 [21].* S. mutans* UA159 biofilms were formed on glass microscope slides (Corning® Incorporated, New York, USA), suspended vertically in Ultrafiltered (10 kDa molecular weight cut-off membrane; Amicon) Tryptone-Yeast Extract Broth (UTYEB) [22], or cation-adjusted-Müller-Hinton broth (Sigma-Aldrich Co., St. Louis, MO, USA) with or without lysed horse blood (MHS or MHS, resp.) [21], all containing 1% sucrose, at 37°C, 10% CO_2_ for 5 days. After 48 h one group of biofilm (baseline 48 h) was collected and the other groups were treated by 1 min, twice a day, with the respective solutions, for 3 days: group 2, 0.9% NaCl (Baseline 120 h) (*n* = 4); group 3, CHX 1 *μ*g/mL (0.0001%) (*n* = 4); group 4, CHX 10 *μ*g/mL (0.001%) (*n* = 4); group 5, CHX 120 *μ*g/mL (0.012%) (*n* = 4); group 6, CHX 1.200 *μ*g/mL (0.12%) (*n* = 4). The biomass and viable bacteria of biofilms were determined and the culture medium pH was determined daily, as an indicator of biofilm acidogenicity. The determination of the pH medium of groups 2 to 6 was carried out directly on the plate, with a calibrated pH meter and controlled environment.

### 2.2. Glass Slides Preparation

**Figure 1:**
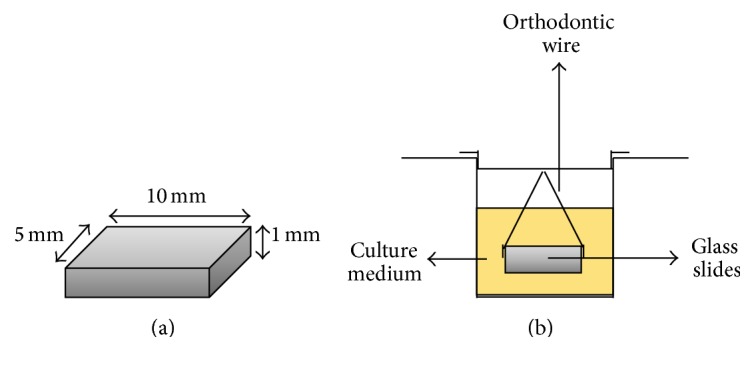
(a) Glass microscope slides. (b) Glass microscope slides on medium culture in the culture plates.

Glass microscope slides (Corning Incorporated, New York, USA) were cut to obtain slides with 10 × 5 × 1 mm (Figure 1(a)). Glass slides were autoclaved, anchored vertically with metal devices, and suspended in a 24-well culture plate (Figure 1(b)).

### 2.3. Biofilm Growth

Ultrafiltered Tryptone-Yeast Extract Broth (UTYEB) [22] or MH or MHS (CLSI M7-A6) [21] was used as culture medium as described as follows.

When UTYEB medium was used,* S. mutans* UA159 colonies (5–10) were transferred to UTYEB containing 1% glucose and incubated for 18–24 h, at 37°C and 10% CO_2_ to reactivate the microorganisms. 100 *μ*L of the suspension was mixed with 50 mL of UTYEB containing 1% sucrose to obtain a final inoculum concentration of 5 × 10^5^ CFU/mL (colony forming unit/milliliter) [23].

When the MH medium was used (with or without blood),* S. mutans* UA159 colonies were transferred for a blood agar plate and it was incubated by 18–24 h, at 37°C and 10% CO_2_ adjusted to a McFarland 0.5 to 0.63 in saline solution, 0.9% corresponding to 5 × 10^8^ CFU/mL. 125 *μ*L of this inoculum was added to 50 mL of MH containing 1% sucrose, in order to obtain a final concentration of 5 × 10^5^ CFU/mL, as recommended by CLSI M7-A6 [21].

The glass slides were individually positioned in wells containing 2.0 mL of the inoculum and were incubated at 37°C and 10% CO_2_ to allow bacterial adhesion. On the next day, the biofilms formed on glass slides were transferred to fresh medium containing 1% sucrose. This procedure was repeated for the next 3 days. The pH of the culture medium was determined daily as an indicator of biofilm acidogenicity.

### 2.4. Treatments

CHX solutions were prepared from 20% chlorhexidine digluconate (Sigma-Aldrich Co., St. Louis, MO, USA) in sterilized distilled water to obtain concentrations of 0.0001, 0.001, 0.012, and 0.12% CHX, since it was previously verified that the CHX's Minimum Inhibitory Concentration (MIC) against* S. mutans* UA 159 is 1 *μ*g/mL (0.0001%) (CLSI M7-A6, 2003 [21]) (unpublished results). Treatments were performed twice a day and after each treatment, the biofilms formed on glass slides were washed 3 times in 0.9% NaCl.

### 2.5. Biofilm Collection

After the assigned experimental time of biofilm growth, the microscope glass slides containing biofilms were washed 3 times in 0.9% NaCl and individually transferred to microcentrifuge tubes containing 1 mL of 0.9% NaCl.

The tubes were sonicated at 7 W for 30 s (Branson, Sonifier 50, Danbury, CT, USA) to detach the biofilms formed on the microscope glass, according to Aires et al. [24]. The glass slides were carefully removed from the suspension and discarded. Aliquots of the suspension were used to determine biofilm bacterial viability and biomass (dry weight).

### 2.6. Biomass Determination

Biofilm dry weight was determined according to Koo et al. [22] from 200 *μ*L of the suspension. For the dry weight determination, three volumes of cold ethanol (−20°C) were added to cell suspension, and the resulting precipitate was collected (10 000 g for 10 min, 4°C). The supernatant was discarded, and the cell pellet was washed twice with cold ethanol, dried, and had the weight checked.

### 2.7. Bacterial Viability

An aliquot of 100 *μ*L of the suspension formed after sonication was diluted in 0.9% NaCl in series up to 10^−7^ and 2 drops of 20 *μ*L of each dilution were inoculated on BHI agar (BD, Sparks, USA) to determine the number of viable microorganisms and assess the successfulness of the sonication procedure [25]. The plates were incubated for 24 h at 37°C and 10% CO_2_ (IG 150, Jouan incubator). CFU were counted and the results were expressed as CFU/mg of biofilm dry weight [24].

### 2.8. Statistical Analysis

The descriptive analysis, in statistical analysis, was carried out presenting measures of position and dispersion for continuous variable. The level of significance adopted for the statistical tests was 5%. ANOVA's test was used to compare the parameters, considering the factors used. The variables were transformed into ranks due to the sample size. Multiple comparisons had been carried through by the test of Tukey and the test of profile for contrasts. The Jonckheere-Terpstra test was used for comparing numerical measures among groups with ordinance and magnitude (tendency).

Capital letters indicate statistical differences between culture media (UTYEB, MH, and MHS), and lowercase letters indicate statistical differences according to the treatments using each culture medium (UTYEB, MH, or MHS), by ANOVA and Tukey tests (*p* < 0.05). The software SPSS for Windows 10.0 (SPSS, Chicago, IL, USA) was used for statistical analysis.

## 3. Results

A statistically significant tendency effect (*p* ≤ 0.002) was found among CHX concentration, biofilm dry weight, and viable bacteria, showing dose-response effect on these variables using UTYEB, MH, or MHS. It was observed that although the amount of biofilm and viable microorganisms formed on glass microscope slides using MH medium was lower, blood did not result in difference between them (MH or MHS).

**Figure 2:**
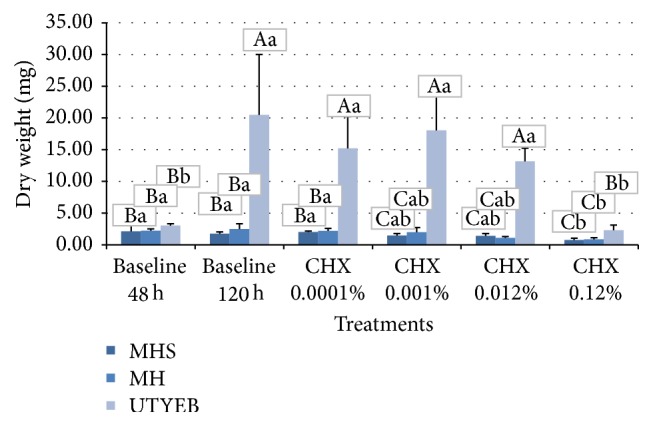
% means and standard deviations of dry weight in the biofilms growth according to the treatments. Capital letters indicate statistical differences between culture media, and lowercase letters indicate statistical differences according to the treatments using each culture medium, by Tukey test (*p* < 0.05).

Biofilms formed on MH or MHS medium presented lower dry weight values than UTYEB (*p* < 0.0001). Using UTYEB, only the dry weight of the biofilm treated with 0.12% CHX had lower values than other groups and same values as baseline 48 h (*p* < 0.0001). Using MH or MHS, lower values of dry weight were found in the groups 0.12% CHX or baseline 48 h than in groups treated with 0.0001% CHX or baseline 120 h. However, in groups treated with 0.012% CHX, 0.001% did not differ from others (*p* = 0.0005) (Table 1 and Figure 2).

**Figure 3 fig3:**
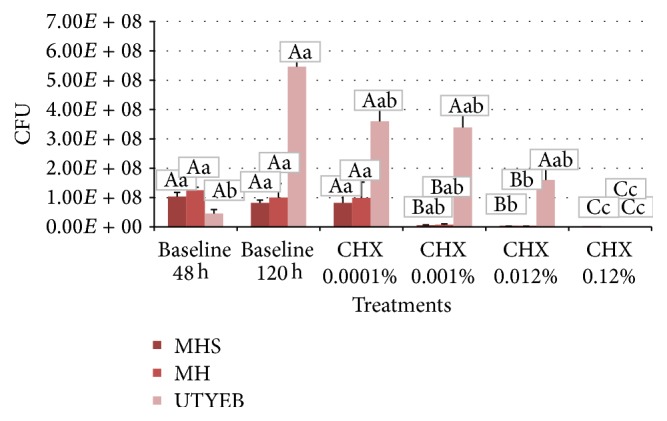
% means and standard deviations of viable bacteria (CFU) in the biofilms growth according to the treatments. Capital letters indicate statistical differences between culture media, and lowercase letters indicate statistical differences according to the treatments using each culture medium, by Tukey test (*p* < 0.05).

Concerning the viable bacteria (UFC), biofilms formed on MH or MHS medium presented lower values than UTYEB (*p* < 0.0001). Using UTYEB, viable bacteria of the biofilm treated with 0.12% CHX had lower values than baseline 48 h group; however, these baselines did not differ from the groups treated with 0.012%, 0.001%, and 0.0001% CHX. Baseline 120 h group presents higher values (*p* < 0.0001). Using MH or MHS, lower values of UFC were found in the group 0.12%, 0.012% CHX, or baseline 48 h compared to those in groups treated with 0.0001% CHX or baseline 120 h. Yet, the groups treated with 0.001% CHX did not differ from the others (*p* < 0.0001) (Table 2 and Figure 3).

**Figure 4 fig4:**
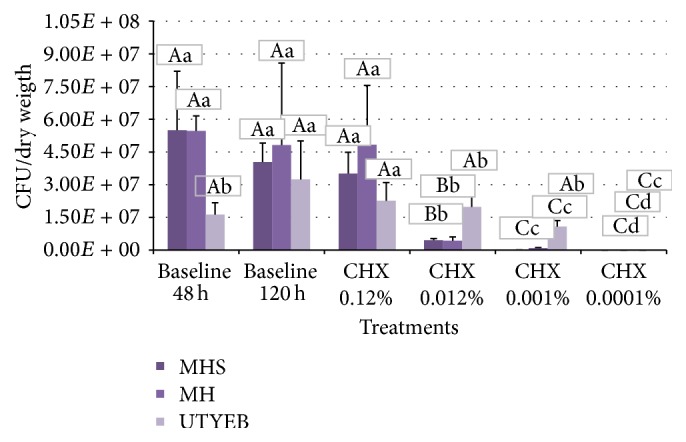
% means and standard deviations of CFU/mg dry weight in the biofilms grown according to the treatments. Capital letters indicate statistical differences between culture media, and lowercase letters indicate statistical differences according to the treatments using each culture medium, by Tukey test (*p* < 0.05).

**Figure 5 fig5:**
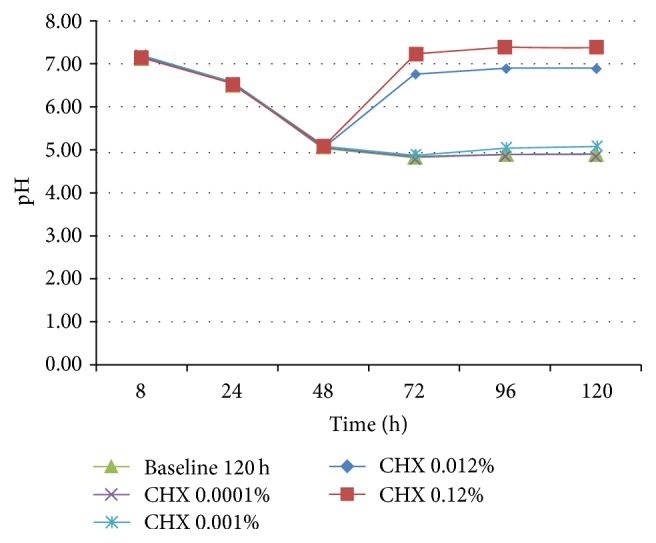
pH of the MH culture medium after 48 h of biofilm growth in the absence of treatments and at each 24 h after starting treatments (72, 96, and 120 h) (*n* = 4).

**Figure 6 fig6:**
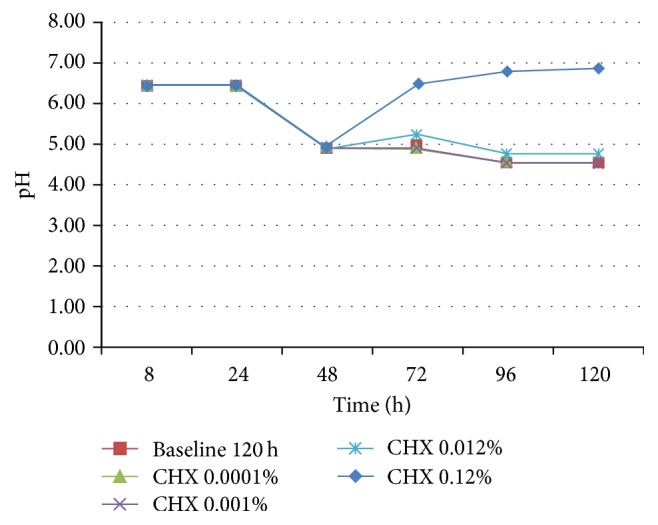
pH of the MHS culture medium after 48 h of biofilm growth in the absence of treatments and at each 24 h after starting treatments (72, 96, and 120 h) (*n* = 4).

**Figure 7:**
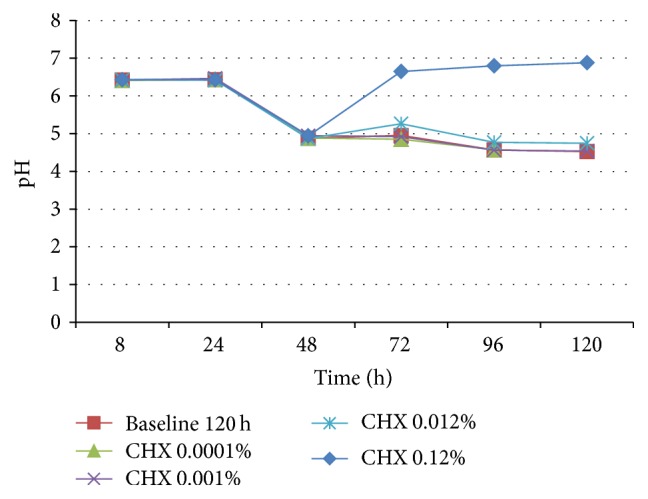
pH of the UTYEB culture medium after 48 h of biofilm growth in the absence of treatments and at each 24 h after starting treatments (72, 96, and 120 h) (*n* = 4).

MH medium, with or without blood, was more sensitive to show biofilm differences in the viable bacteria/mg dry weight, when compared to protocol using UTYEB medium (Table 3 and Figure 4), decreasing the biofilm capacity to produce acids (Figures 5, 6, and 7). Using UTYEB the concentration of 0.012% CHX or lower had a bacteriostatic effect, not interfering with the dry weight and viable bacteria counts, but affecting the acid production level, which was lower than that of the control group.

Biofilms formed on MH or MHS medium presented lower values of the biomass (viable microorganisms/mg dry weight) than UTYEB (*p* < 0.0001). Using UTYEB, the biomass of biofilm treated with 0.12% CHX showed lower values than other groups, and baseline 120 h presented the higher values. Using MH or MHS, lower values were found in the groups treated with 0.12% CHX, which was lower than 0.012% CHX group and this was lower than 0.001% CHX group. The group treated with 0.0001% CHX and baselines 48 h and 120 h presented higher values than the others (*p* < 0.0001) (Table 3).

At all times, using MH, with or without blood (Figures 5 and 6), group 0.12% CHX showed higher pH than group 0.012% CHX, which showed higher pH than 0.0010% CHX, and those groups presented higher pH than other groups. However, using UTYEB, only the group 0.12% CHX showed higher pH than group 0.012% CHX and those groups presented higher pH than others (Figure 7).

## 4. Discussion

The polymicrobial community of dental biofilm is one of the best-studied kinds of biofilms. When it comes to oral biofilm, more than 500 species or phylotypes have been identified [26]. Dental biofilm is challenged by frequent changes in environmental conditions, for example, food intake, temperature, pH change, and salivary flow. Perhaps as a response to environmental challenges, the oral biofilm community has evolved with individual members assuming specialized functions, for example, primary and secondary colonizers [27], including members that can metabolize excreted products (such as lactic acid) produced by other species [28].

It is challenging to control diseases caused by biofilms due to the difficulty in finding substances able to interfere with factors involved with bacterial organization in a biofilm, as well as the antibacterial properties of the biofilm structure itself [29]. Antiplaque agents are designed to (a) prevent the formation of the biofilm and/or (b) remove established biofilm. In contrast, the mode of action of antimicrobial agents involves inhibiting the growth or killing the target bacteria, expressed in terms of their MIC or MBC, respectively [30].

There are currently a variety of model systems available that could be applied for studying the process of human dental caries, each of these showing advantages and disadvantages. An* in vitro* model system using bacterial biofilms is likely to display less inherent variability than an* in situ* model system, since variables such as fluid (saliva) flow, carbohydrate intake, and bacterial population composition can be controlled more accurately* in vitro* [31]. The model of* S. mutans* biofilm growth tested in this study, described by Koo et al. [22] and modified by Ccahuana-Vásquez and Cury [23] using MH, as indicated by CLSI M7-A6 [21], was validated and the dose-response effect of CHX on* S. mutans* biofilm was shown for all variables.

The main relevance of this study is that it used the standards of the Clinical and Laboratory Standards Institute (CLSI, formerly NCCLS), an international, interdisciplinary, nonprofit, standards-developing, and educational organization that promotes the development and use of voluntary consensus standards and guidelines within the healthcare community. It is recognized worldwide for developing standards and guidelines for patient testing and related healthcare issues. Their process is based on the principle that consensus is an effective and cost-effective way to improve patient testing and health care services. In addition to developing and promoting the use of voluntary consensus standards and guidelines, they provide an open and unbiased forum to address critical issues affecting the quality of patient and health care (CLSI M7-A6 [21]).

In the present study, the model using CLSI M7-A6 [21] protocol showed a lower amount of biofilm and viable microorganisms formed on glass microscope slides and was more sensitive than the model described by Koo et al. [22] and modified by Ccahuana-Vásquez and Cury [23] in showing biofilm changes in the presence of antimicrobial substances. These differences can be supported by the Shemesh et al. [20] study, which tested some nutrient components and their influence on gene expression in* S. mutans* biofilms under various conditions.

Using differential analysis of the transcripts from* S. mutans* growth in media of various nutrient contents they observed the pivotal role of the dietary in the pathogenicity of* S. mutans* biofilm and the influence of such carbohydrates on gene expression, which can present different responses to antimicrobial substances, as well as on biofilm thickness. For both media, the treatment twice a day with 0.12% CHX showed a bactericidal effect, avoiding the increase in biofilm mass (dry weight) (Table 1 and Figure 2) and eliminating a large proportion of the viable bacteria in the biofilm (Table 2 and Figure 3). However, using MH, which has nutrient content recognized and approved by CLSI M7-A6 [21], the concentration of 0.012% CHX interfered with the dry weight and viable bacteria counts and affected the acid production level, which was lower than that in the control group [32] (Tables 1 [Table tab2]–3) (Figures 2 [Fig fig3] [Fig fig4] [Fig fig5] [Fig fig6]–7).

Its mechanism of action includes direct damage to the internal cytoplasmatic membrane, being bacteriostatic at low dosages and bactericidal at high concentrations [15]. This implies that the structural damage caused by 0.012% CHX was less than that caused by 0.12% CHX or lower concentrations. The results found with the use of both models that were supported by a clinical trial show that 0.12% CHX is more effective in reducing S. mutans CFU than lower concentrations [33]. In spite of that, more studies are necessary to evaluate the influence of a culture medium on dose-response effect of the chlorhexidine (CHX) on* Streptococcus mutans* UA159 biofilm, formed on enamel slabs, and enamel demineralization.

## 5. Conclusions

We can conclude that the culture medium does influence dose-response effect of chlorhexidine (CHX) on* Streptococcus mutans* biofilm. The results suggest that MH broth can be used as an alternative medium for antibacterial activity without blood supplementation.

## Figures and Tables

**Table 1:** % means and standard deviations of dry weight in the biofilms growth according to the treatments.

Groups	Dry weight (mg)		
	MH	MHS	UTYEB
Baseline 48 h	2.13 (1.03)^Ba^	2.25 (0.29)^Ba^	3.05 (0.26)^Bb^
Baseline 120 h	1.75 (0.29)^Ba^	2.50 (0.82)^Ba^	20.49 (9.51)^Aa^
CHX 0.0001%	2.00 (0.18)^Ba^	2.23 (0.39)^Ba^	15.20 (5.02)^Aa^
CHX 0.001%	1.48 (0.31)^Cab^	1.98 (0.74)^Cab^	18.04 (5.42)^Aa^
CHX 0.012%	1.43 (0.37)^Cab^	1.10 (0.23)^Cab^	13.19 (2.05)^Aa^
CHX 0.12%	0.75 (0.29)^Cb^	0.88 (0.25)^Cb^	2.30 (0.82)^Bb^

Means within lines followed by distinct capital letters and those in columns followed by lower case letters differ statistically by Tukey test (*p* < 0.05).

**Table 2 tab2:** % means and standard deviations of viable bacteria (CFU) in the biofilms growth according to the treatments.

Groups	CFU		
	MH	MHS	UTYEB
Baseline 48 h	1.03*E + *08 (1.44*E + *07)^Aa^	1.25*E + *08 (1.00*E + *07)^Aa^	4.50*E + *07 (1.41*E + *07)^Ab^
Baseline 120 h	8.17*E + *07 (1.01*E + *07)^Aa^	1.00*E + *08 (4.77*E + *07)^Aa^	5.46*E + *08 (5.89*E + *07)^Aa^
CHX 0.0001%	8.17*E + *07 (2.93*E + *07)^Aa^	9.83*E + *07 (5.43*E + *07)^Aa^	3.60*E + *08 (4.34*E + *07)^Aab^
CHX 0.001%	5.67*E + *06 (8.78*E* *+* 05)^Bab^	7.42*E + *06 (2.93*E + *06)^Bab^	3.39*E + *08 (4.16*E + *07)^Aab^
CHX 0.012%	8.92*E + *05 (8.04*E + *04)^Bb^	9.17*E + *05 (3.82*E + *04)^Bb^	1.60*E + *08 (5.88*E + *07)^Aab^
CHX 0.12%	8.33*E + *02 (2.89*E + *02)^Cc^	8.58*E + *02 (8.43*E + *02)^Cc^	1.58*E + *02 (5.20*E + *01)^Cc^

Means within lines followed by distinct capital letters and those in columns followed by lower case letters differ statistically by Tukey test (*p* < 0.05).

**Table 3:** % means and standard deviations of CFU/mg dry weight in the biofilms grown according to the treatments.

Groups	CFU/dry weight		
	MH	MHS	UTYEB
Baseline 48 h	5.50*E* + 07 (2.70*E* + 07)^Aa^	5.47*E* + 07 (6.82*E* + 06)^Aa^	1.63*E* + 07 (5.36*E* + 06)^Ab^
Baseline 120 h	4.04*E* + 07 (8.72*E* + 06)^Aa^	4.82*E* + 07 (3.76*E* + 07)^Aa^	3.24*E* + 07 (1.76*E* + 07)^Aa^
CHX 0.0001%	3.51*E* + 07 (9.75*E* + 06)^Aa^	4.84*E* + 07 (2.72*E* + 07)^Aa^	2.26*E* + 07 (8.37*E* + 06)^Ab^
CHX 0.001%	4.52*E* + 06 (7.75*E* + 05)^Bb^	4.28*E* + 06 (1.74*E* + 06)^Bb^	1.98*E* + 07 (6.18*E* + 06)^Ab^
CHX 0.012%	7.94*E* + 04 (1.07*E* + 04)^Cc^	8.65*E* + 05 (2.29*E* + 05)^Cc^	1.07*E* + 07 (2.79*E* + 06)^Ab^
CHX 0.12%	1.03*E* + 03 (7.76*E* + 02)^Cd^	1.02*E* + 03 (7.59*E* + 02)^Cd^	1.20*E* + 03 (2.23*E* + 03)^Cc^

Means within lines followed by distinct capital letters and those in columns followed by lower case letters differ statistically by Tukey test (*p* < 0.05).
